# Role of Stent Versus Thrombolysis in Management of Cocaine-Induced ST-Elevation Myocardial Infarction

**DOI:** 10.7759/cureus.9654

**Published:** 2020-08-11

**Authors:** Zin Thawdar Oo, Htoo Kyaw

**Affiliations:** 1 Internal Medicine, LaSante Health Center, Brooklyn, USA; 2 Cardiology, Mount Sinai Hospital, New York, USA

**Keywords:** cocaine induced myocardial infarction, non-cardiac chest pain, pci, st-elevation myocardial infarction (stemi), coronary revascularization

## Abstract

Cocaine has been used increasingly nowadays because of its abundant availability and recreational effects. Along with that, we have been experiencing many cases presenting with cocaine intoxication and withdrawal effects, including hypertension and chest pain syndrome. In the modern era, many medical advances related to myocardial infarction treatment have been made, including not only medical therapy but also urgent or emergent reperfusion, and revascularization therapies. In percutaneous coronary revascularization therapy, a second-generation drug-eluting stent (DES) with dual antiplatelet therapy is the first-line treatment compared to bare-metal stent (BMS) with significant reduced risk of stent thrombosis and restenosis. However, there is limited clinical and research data on how to approach cocaine-induced myocardial infarction (CIMI) and it remains unclear what would be the optimal stent type we should use in CIMI. We, hereby, would like to describe a case of cocaine-related ST-segment elevation myocardial infarction (STEMI) requiring emergent percutaneous coronary intervention with a DES and clinical outcome. We also performed a literature review of cocaine-induced acute myocardial infarction management.

## Introduction

Cocaine is derived from the Erythroxylum coca plant and categorized as a Schedule II medication under the Controlled Substances Act in the United States [1]. Cocaine was used globally by 18 million people, representing 0.4% of the adult population aged between 15 and 64 years, along with increased global cultivation of coca bush and cocaine manufacture by 15% and 25%, respectively, in 2017 [2]. Of all cocaine-related medical problems, the impact on the cardiovascular system is regarded as one of the most severe sequelae and the number of chest pain patients admitted to hospitals is increasing steadily because of its effects on the heart and blood vessels resulting in coronary artery vasospasm and ischemia. Other cocaine-induced myocardial infarction (CIMI) complications, including congestive heart failure, ventricular tachycardia, supraventricular tachycardia, and bradyarrhythmia, can manifest mostly within 12 hours of presentation, but the mortality rate of hospitalized CIMI patient is surprisingly low [3]. However, definitive management for CIMI is still controversial.

## Case presentation

**Figure 1:**
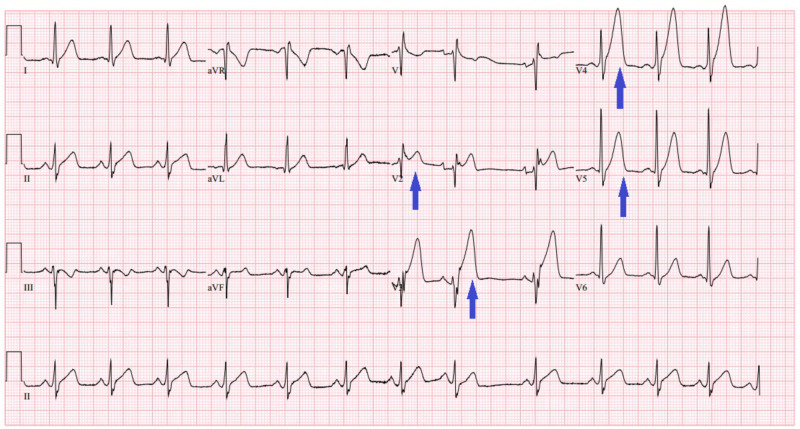
Electrocardiogram showing anteroseptal ST elevation and hyperacute T wave in lateral leads.

A 42-year-old male patient presented to the emergency room with a sudden onset of retrosternal, pressure-like, chest pain. Emergency medical service (EMS) was called in advance for ST-segment elevation myocardial infarction (STEMI) alert as the electrocardiogram (EKG) showed a 3-mm STEMI in the anterolateral leads with hyperacute T waves in the lateral leads (Figure 1). Initial vital signs were blood pressure of 149/90 mmHg, pulse of 78 bpm, respiratory rate of 15 per minute, and oxygen saturation of 99%. He had no other medical problems but disclosed cocaine use approximately four hours before his presentation. He also confessed that he was an occasional cocaine user.

**Figure 2:**
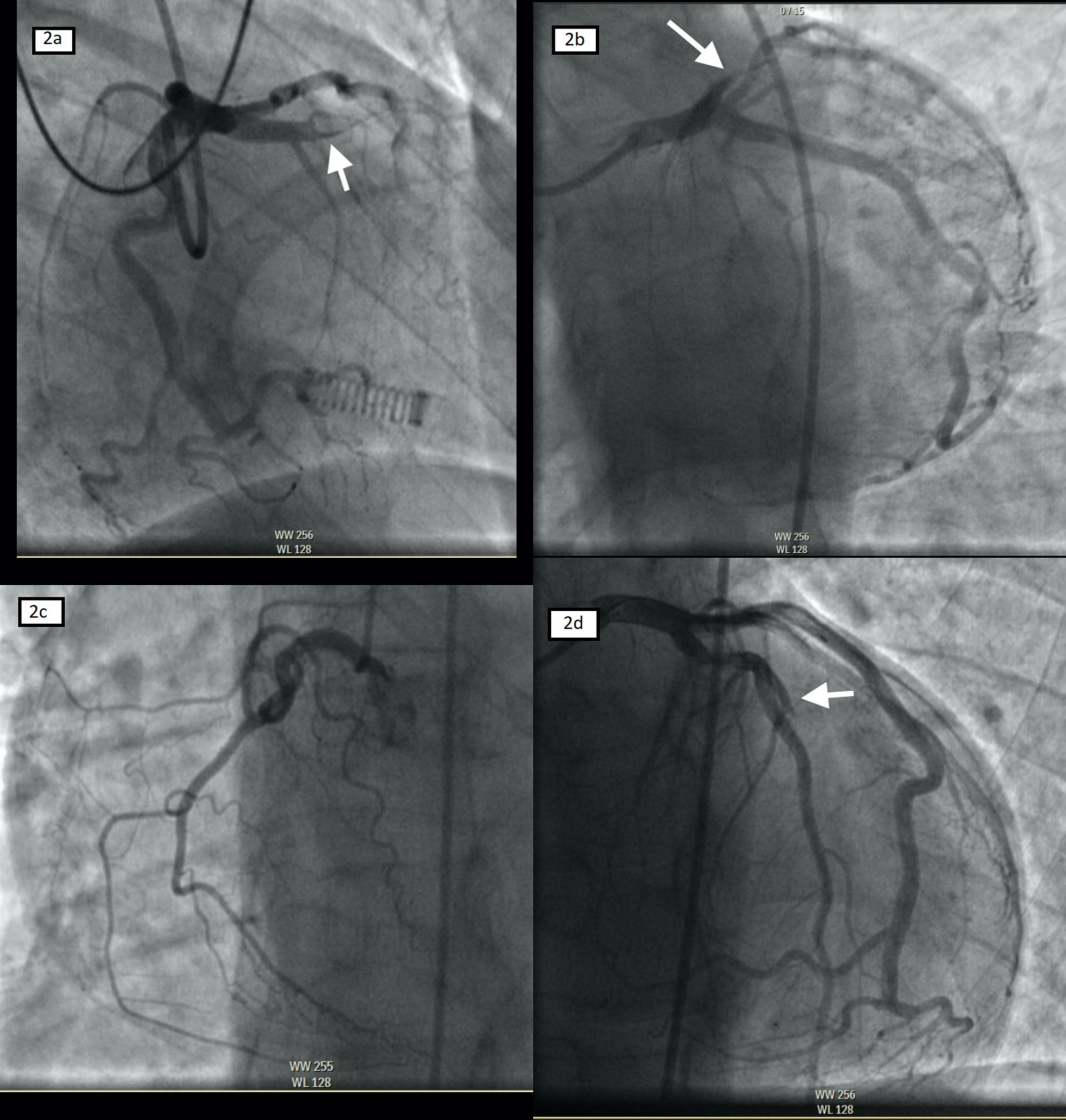
Coronary angiogram showing an acute thrombotic lesion (white arrow) with TIMI 0 flow in the proximal LAD [RAO caudal (a) and LAO caudal views (b)] and normal right coronary artery [LAO view (c)]. After percutaneous coronary intervention with drug-eluting stent placement, there was TIMI 3 flow in the LAD artery with distal embolization of diagonal artery (white arrow) [AP cranial view (d)]. LAD = left anterior descending artery; TIMI = thrombolysis in myocardial infarction; RAO = right anterior oblique; AP = anteroposterior; LAO = left anterior oblique.

Aspirin 325 mg, ticagrelor 180 mg, atorvastatin 80 mg, and heparin 5,000 mg IV bolus were given immediately, and he was brought to the cardiac catheterization lab for emergent angiography which revealed thrombotic occlusion of middle left anterior descending (LAD) artery (Figure 2). Percutaneous coronary intervention (PCI) with a drug-eluting stent (DES) was successfully placed (Figure 2). Immediately after DES placement, the patient had episodes of ventricular fibrillation requiring four DCCV (direct current cardioversion) with an amiodarone 300 mg bolus to convert to sinus rhythm. Further angiographic images showed a thrombotic occlusion in the first diagonal artery but no further intervention was done as the patient’s chest pain had resolved with thrombolysis in myocardial infarction (TIMI) 3 flow in LAD. The patient was sent to the cardiac care unit for further medical optimization. Intravenous amiodarone infusion was given for 24 hours and subsequently stopped as no further arrhythmia events were noted on the telemetry monitoring. Lab tests revealed peaked troponin of 10.5 ng/ml with positive cocaine in urine toxicology.

**Figure 3:**
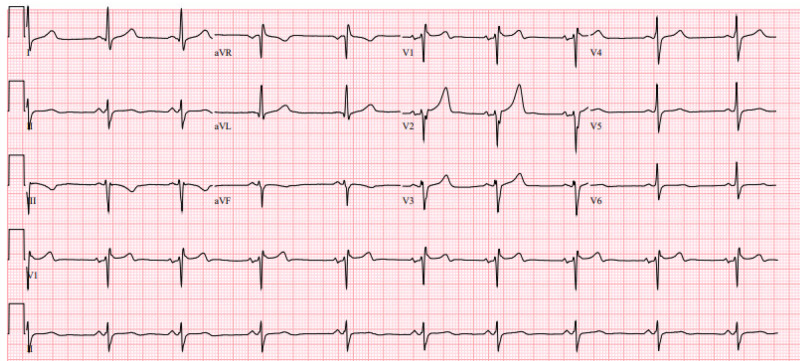
Electrocardiogram showing sinus bradycardia with incomplete right bundle branch block after percutaneous coronary intervention.

Post-intervention EKG showed sinus bradycardia and incomplete right bundle branch block with a resolution of ST elevation (Figure 3). Transthoracic echocardiogram demonstrated mild concentric left ventricular (LV) hypertrophy with mildly reduced LV function (LV ejection fraction 45%-50%). The rest of the hospital stay was uneventful and the patient was prescribed with aspirin 81 mg once daily, ticagrelor 90 mg every 12 hours, and atorvastatin 80 mg once daily upon discharge with outpatient follow-up. 

## Discussion

Cocaine has been used for more than 5,000 years since South America’s Erythroxylum coca plant was discovered, and approximately more than 550,000 emergency visits per year are due to cocaine-related complications. Cocaine can provoke significant hemodynamic changes including coronary artery vasospasm, alteration of coagulation systems resulting in increased thrombogenicity, and a higher risk of myocardial ischemia due to increased myocardial demand [4,5]. As per New York City Department of Health and Mental hygiene, cocaine was the most frequently cited drug in emergency department (ED) visits for all age groups, with 425 cocaine-related visits for every 100,000 New Yorkers. In 2017, more than eight in ten (82%) overdose deaths involved an opioid, followed by heroin and cocaine with 52% and 49%, respectively. Moreover, the rate of overdose deaths in high poverty neighborhoods was two times higher than residents of medium and low poverty neighborhoods. In the United States, the rate of cocaine overdose death has doubled from 2007 to 2017, especially since 2014 [2].

Although we have been dealing with cocaine for millennia, the management of drug-related cardiovascular manifestations remains uncertain. Interestingly, back in the 1990s, cocaine-related chest pain was managed conservatively. Hollander et al. reported that 83 out of 246 patients with CIMI were found to have gotten better with nitroglycerine alone [6]. One prospective study found the early use of lorazepam plus nitroglycerine is more efficacious and safer than nitroglycerine alone with a significant reduction in chest pain score [7].

Based on our literature review, it is astounding to know that the management of CIMI did not have much literature evidence or proper guideline recommendation [8]. Hence, medical therapy, thrombolytic therapy, and PCI have been used based on individual clinical situation and providers' experiences. However, there were reports of increased risk of intracranial hemorrhage and oropharyngeal bleeding after the use of thrombolytic therapy in CIMI [9]. Moreover, factors associated with poor health, such as poor access to medical care, medication noncompliance, loss to follow-up, and repeated unhealthy behaviors remain major deciding factors for the initial therapeutic approach [10]. American College of Cardiology/American Heart Association (ACC/AHA) scientific statement from 2013 recommended a timely PCI by experienced operators rather than thrombolytic therapy in those presenting with STEMI (Class of recommendation I) [8]. Intravenous substance abusers also have a higher risk of having mycotic aneurysm because of its local trauma, infection, or thromboembolic phenomenon, and thus the use of thrombolytic therapy can be limited by enhancing the bleeding risk of the mycotic aneurysm which can lead to the adverse neurological deficit and intracranial hemorrhage [11].

Kasim et al. reported a case of cocaine-associated STEMI, and the angiogram showed thrombus in the left main coronary artery with a partially occluded dominant left circumflex with TIMI 3 flow. The patient was treated with intravenous abciximab along with seven days of enoxaparin, and repeated angiogram showed resolution of the thrombus [12]. Our patient was required to undergo emergent cardiac catheterization with PCI to LAD. While PCI with DES has improved the landscape of acute coronary syndrome management, the risk of stent thrombosis in cocaine users has significantly increased, partly due to medication noncompliance, heterogeneity of beta-blocker usage, repeated use of cocaine, and increased thrombogenicity [13,14].

McKee et al. showed that stent thrombosis had occurred in 7.6% of cocaine abusers compared to the control group (0.6%) during the nine-month follow-up period [13]. Ibrahim et al. illustrated the case of unstable angina that underwent PCI to the LAD artery with bare-metal stent (BMS). Three-day later, the patient came back with subacute stent thrombosis while on dual antiplatelet therapy and underwent emergent manual aspiration as well as AngioJet mechanical thrombectomy with balloon angioplasty [15]. Although the risk of stent-related complications is undeniably present in acute coronary syndrome with the cocaine-use disorder (CUD), the decision whether to use stent or no stent strategy is more complex and delicate than saying “yes or no”.

Moreover, there is another uncertain area of medical therapy in those with CUD. The utility of beta-blocker in cocaine-associated myocardial infarction is always controversial because of its theoretical risk of unopposed alpha-adrenergic action resulting in exaggerated vasospasm with uncontrolled hypertension. However, we have no real-world clinical data of cocaine-related vasospasm and hypertension [4]. Banerji et al. published the safety data of carvedilol usage among 2,578 individuals with heart failure and CUD. It showed that carvedilol was safe to use in these populations without any adverse outcomes [16].

We have learned a great deal regarding cardiac catheterization and PCI in the past years, which is a life-saving procedure for STEMI, but the safety and efficacy of stent placement in STEMI with CUD remain unclear with limited literature evidence. Hence, the clinical decision regarding plain old balloon angioplasty, balloon angioplasty +/- stent placement, or thrombolytic therapy should be made based on the pertinent clinical history and individualized factors. Ultimately, lifestyle modification with the prevention of cocaine use is paramount to have the best clinical outcomes.

## Conclusions

Management of STEMI associated with cocaine use remains an area that requires further investigation. Our patient underwent urgent PCI with DES without any complications. Although there is no clear consensus about direct stenting in all cocaine-related acute myocardial infarction, this case highlights the beneficial effect of PCI and supports the judicious use of PCI along with optimal medical therapy.

## References

[1] Drug Enforcement Administration (2020). Drugs of Abuse, A DEA Resource Guide. D.

[2] United Nations Office on Drugs and Crime (2019). World Drug Report 2019. Report no. E.19.XI.8. https://wdr.unodc.org/wdr2019/prelaunch/WDR19_Booklet_4_STIMULANTS.pdf.

[3] Hollander JE, Hoffman RS, Burstein JL, Shih RD, Thode HC Jr (1995). Cocaine-associated myocardial infarction. Mortality and complications. Cocaine-Associated Myocardial Infarction Study Group. Arch Intern Med.

[4] Boehrer JD, Moliterno DJ, Willard JE (1992). Hemodynamic effects of intranasal cocaine in humans. J Am Coll Cardiol.

[5] Rezkalla SH, Mazza JJ, Kloner RA, Tillema V, Chang SH (1993). Effects of cocaine on human platelets in healthy subjects. Am J Cardiol.

[6] Hollander JE, Hoffman RS, Gennis P (1994). Nitroglycerin in the treatment of cocaine associated chest pain: clinical safety and efficacy. J Toxicol Clin Toxicol.

[7] Honderick T, Williams D, Seaberg D, Wears R (2003). A prospective, randomized, controlled trial of benzodiazepines and nitroglycerine or nitroglycerine alone in the treatment of cocaine-associated acute coronary syndromes. Am J Emerg Med.

[8] O'Gara PT, Kushner FG, Ascheim DD (2013). 2013 ACCF/AHA guideline for the management of ST-elevation myocardial infarction: executive summary: a report of the American College of Cardiology Foundation/American Heart Association Task Force on Practice Guidelines. Circulation.

[9] Hollander JE, Wilson LD, Leo PJ, Shih RD (1996). Complications from the use of thrombolytic agents in patients with cocaine associated chest pain. J Emerg Med.

[10] Ho PM, Spertus JA, Masoudi FA (2006). Impact of medication therapy discontinuation on mortality after myocardial infarction. Arch Intern Med.

[11] Bush HS (1988). Cocaine-associated myocardial infarction. A word of caution about thrombolytic therapy. Chest.

[12] Kasim S, O'Donabhain R, Mcfadden E (2011). Cocaine-associated myocardial infarction: should they all be stented?. Case Rep Cardiol.

[13] McKee SA, Applegate RJ, Hoyle JR, Sacrinty MT, Kutcher MA, Sane DC (2007). Cocaine use is associated with an increased risk of stent thrombosis after percutaneous coronary intervention. Am Heart J.

[14] Karlsson G, Rehman J, Kalaria V, Breall JA (2007). Increased incidence of stent thrombosis in patients with cocaine use. Catheter Cardiovasc Interv.

[15] Ibrahim M, Hasan R, Awan M (2013). Cocaine-induced coronary stent thrombosis. Exp Clin Cardiol.

[16] Banerji D, Alvi RM, Afshar M (2019). Carvedilol among patients with heart failure with a cocaine-use disorder. JACC Heart Fail.

